# The Scar-suppressing Efficiency of Three Sutures with Different Degradation Rates: A Prospective Split-Scar Study

**DOI:** 10.1007/s00266-025-05288-8

**Published:** 2025-10-23

**Authors:** Xinxi Zhu, Yifan Qiao, Wenbo Liu, Jinyuan Zhu, Hailong Shen, Yuanmei Huang, Rongguang Lai, Gengrui Nan, Maoguo Shu, Jing Jia

**Affiliations:** 1 Department of Plastic, Cosmetic and Maxillofacial, The First Affiliated Hospital of Xi’an Jiaotong University, No. 277, Yanta West Road, Xi’an, 710061 Shaanxi Province China; 2 Department of Medical Oncology, The First Affiliated Hospital of Xi’an Jiaotong University, Xi’an, Shaanxi China; 3 Key Laboratory of Shaanxi Province for Craniofacial Precision Medicine Research & Clinical Research Center of Shaanxi Province for Dental and Maxillofacial, Xi’an, Shaanxi China; 4 Department of Stomatology, Jingbian county People’s Hospital, Yulin, Shaanxi China; 5 Xi’an Jiaotong University, Xi’an, Shaanxi China

**Keywords:** Suture, Degradation rate, Scar suppression, Tension reduction, Inflammation

## Abstract

**Background:**

Our previous study demonstrated that prolonged tension reduction results in satisfactory scar suppression during modified intradermal suturing. The type of suture used in intradermal sutures is crucial for tension preservation, wherein suture degradation results in tension reduction. However, evidence revealing the optimal suture to confront local tension for a prolonged period of time is lacking.

**Objective:**

To compare the aesthetic outcomes associated with three sutures: polyglactin acid and polydioxanone, which are absorbable sutures with tension-maintaining times of 1 and 3 months, respectively, and the nonabsorbable suture polyester.

**Methods:**

We evenly divided a hypogastric incision into three segments before randomly stitching them with three different sutures. After 1, 3 and 6 months, the aesthetic outcomes of the scars brought by each suture were evaluated by scar assessment scales.

**Results:**

Polyglactin acid had the worst aesthetic outcome at 1, 3 and 6 months. Polydioxanone and polyester had similar aesthetic outcomes at 1 and 3 months. However, at 6 months, polyester showed unsatisfying scar inhibition compared to that of polydioxanone. To explore the underlying mechanism, we repeated the above process in mice and found enhanced inflammation in tissues stitched using polyester. The inflammation neutralized the anti-scarring efficiency of reduced tension.

**Conclusion:**

Our study revealed that prolonged reduction in local tension plays an important role in inhibiting scarring, and the ability of sutures to induce local inflammation cannot be ignored.

**Level of Evidence II:**

This journal requires that authors assign a level of evidence to each article. For a full description of these Evidence-Based Medicine ratings, please refer to the Table of Contents or the online Instructions to Authors www.springer.com/00266

## Introduction

**Fig. 1:**
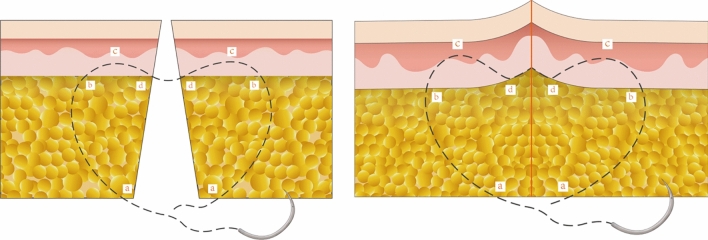
Cross-sectional view depicting the modified buried vertical mattress suture (MBVMS) before (left part) and after (right part) knotting. During the process stitching, stitch moves a long arc track in the dermis in order to recruit as much as possible dermal tissue into the suture loop, resulting in the everting skin and reducing the local tension. At the edge of the incision, the stitch exited from the subcutaneous–cutaneous boundary to avoid a foreign body reaction in dermis

Our previous study demonstrated that wedge excision combined with modified buried vertical mattress suture (WE-MBVMS, Fig. 1) provides better aesthetic outcomes than traditional methods [1, 2]. The absorbable suture used in the WE-MBVMS plays a key role in confronting tension. As the sutures degrade in vivo, the ability of tension reduction weakens or even disappears, which indicates the end of the tension reservation time. The period of scar molding is as long as 6–12 months [3], during which time local tension contributes greatly to scar formation [4]. Hence, selecting appropriate material for the WE-MBVMS to withstand the local tension around the incision might consequently lead to ideal scar suppression. However, surgeons currently select sutures for WE-MBVMS mostly according to their personal preference and clinical experience. Moreover, clinical comparative evidence revealing which suture could be used against the surrounding tension for a long enough time to obtain the optimal cosmetic outcome is lacking.

Currently, various kinds of absorbable sutures can be selected as intradermal sutures, among which polydioxanone (PDS) and polyglactin (GLA) are the most commonly used. With tension holding times of 90 days and 30 days [5], respectively, it is easy for us to relate them to different scar-suppressing abilities. Considering that nonabsorbable sutures, which are also widely used in intradermal sutures, are not absorbed and might provide prolonged dermal support [6, 7], we employed polyester (PE) nonabsorbable sutures in our study. The tension holding time of the polyester remains unclear, but we believe that it will not be permanent. Although the tension-reducing ability of PE will not be disturbed by its degradation in the body, tissue cutting and displacement will occur under the continuous action of external force over time. It follows that the efficiency of tension reduction and tissue eversion associated by PE gradually decreases.

We selected the lower abdominal incision induced by harvesting autogenous skin grafts for wound repair to compare the above three sutures. The donation involves an incision, which leaves a scar at the donor site that is mostly neglected by surgeons but is a concern of patients. In addition, the hypogastrium is an important aesthetic unit; thus, scars are aesthetically undesirable or symptomatic [8]. Moreover, autogenous hypogastric skin grafts, as important skin donor sites, are commonly used by plastic surgeons.

Here, we aimed to establish a feasibility trial comparing the scars left by sutures with different degradation times. By comparing the appearance of wounds/scars achieved by the three sutures over a 6-month follow-up period in a split-scar model, we further clarify the duration of tension reduction required for optimal scar inhibition and provide a reference for suture selection.

## Methods and Analysis

### Study Design

This feasible single-center RCT included 18 patients and compared the aesthetic outcomes of scars sutured by three different-absorption-rate sutures with those of WE-MBVMS. The incision induced by donating skin grafts was evenly divided into three segments, and each segment used one of three different sutures that were randomly allocated by SAS (V.9.4) statistical software. The feasibility of this study was assessed according to the primary outcomes, including patient and clinician enrollment refusal as well as their reasons, reasons for ineligibility, the recruitment ratio, retention and withdrawal at each follow-up point (1, 3, 6 and 12 months), reasons for withdrawal, integrity of collected data and adverse event rates. Secondary outcome measures of the cosmetic outcome of scars will help shape future fully powered RCTs by influencing the sample size.

### Methods and Analysis

**Fig. 2:**
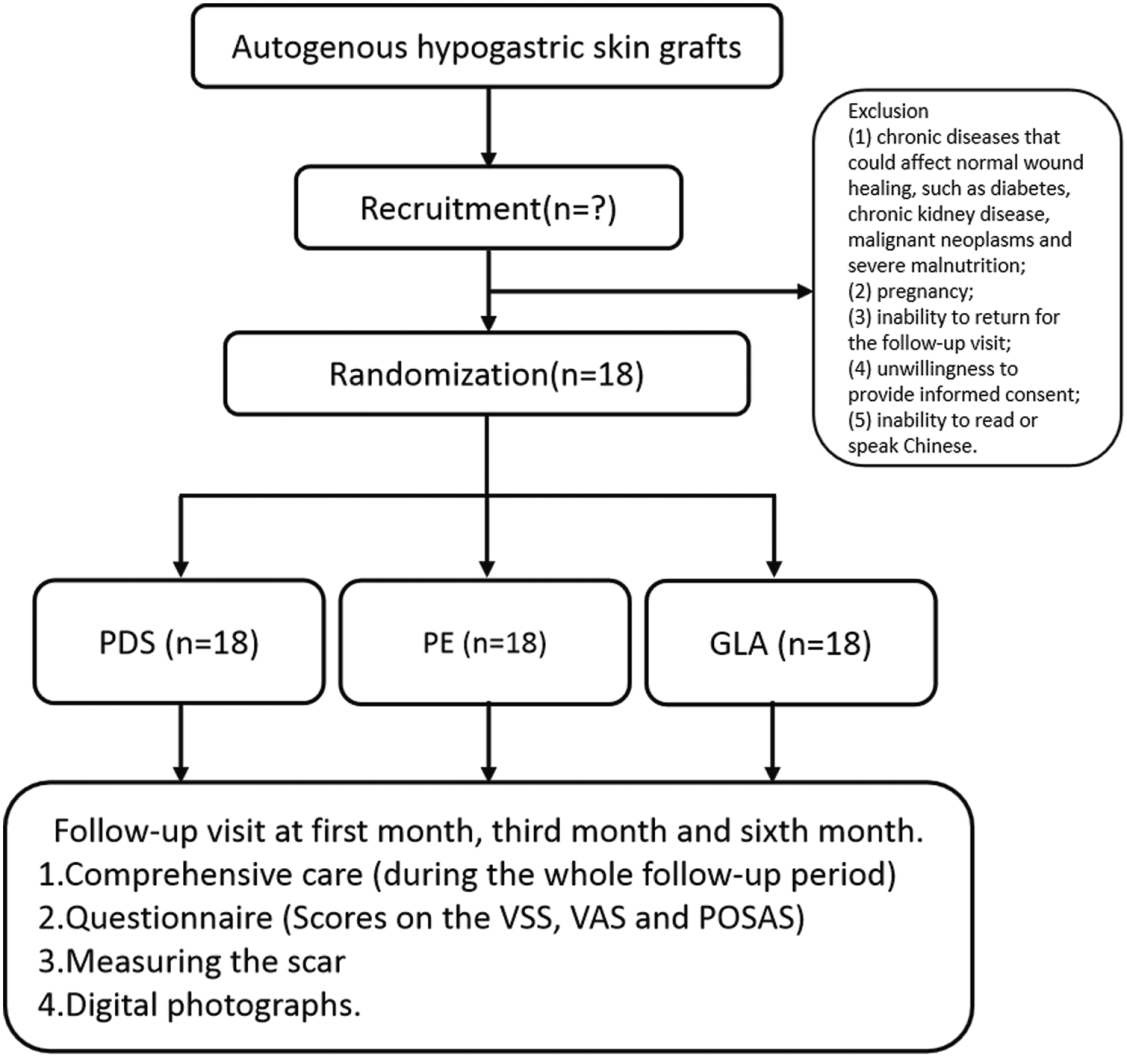
Flow chart depicting the trial

As a single-center prospective RCT, this trial compared the cosmetic outcomes of three sutures with different tension holding times. A hypogastric incision induced by donating skin grafts was chosen for this study. The study was a single-blind study, since the different sutures were sold to the surgeon. However, all the outcome assessors and the subjects were blinded to the treatment assignment. Postoperative follow-ups were performed at 1, 3, 6 and 12 months after surgery. A flowchart of study recruitment, randomization, follow-up and analysis is shown in Fig. 2.

### Recruitment

The participants were recruited from the First Affiliated Hospital of Xi’an Jiaotong University. In a secure, local-area-network-based system, we recorded general information of the recruited patients, including the patient’s name, ID number, residence, sex, age, race, height and weight. Clinicians screened potential participants for eligibility. The participants were not informed about the group to maintain blinding in this regard. They were clearly told that all of the different sutures that we used were appropriate for their condition and the aim of this study. When they decided to participate, they were asked to provide written informed consent, and the research associate provided patients who met the selection criteria with all of the study presentation materials. All participants were registered after ethical approval and registration were obtained. All the above processes followed the ethical principles for medical research involving human subjects of the Declaration of Helsinki, adopted by the 18th General Assembly of the World Medical Association (World Medical Association, 1964).

### Participants

#### Inclusion Criteria

1. Surgical incisions must be closed after harvesting the skin;
2. Incisions at least 10 cm in length;
3. Patients aged between 18 and 60 years.

#### Exclusion Criteria

1. Chronic diseases, such as diabetes, chronic kidney disease, malignant neoplasms and severe malnutrition;
2. Pregnancy;
3. Inability to return for the follow-up visit;
4. Unwillingness to provide informed consent;
5. Inability to read or speak Chinese.

### Randomization

**Fig. 3:**
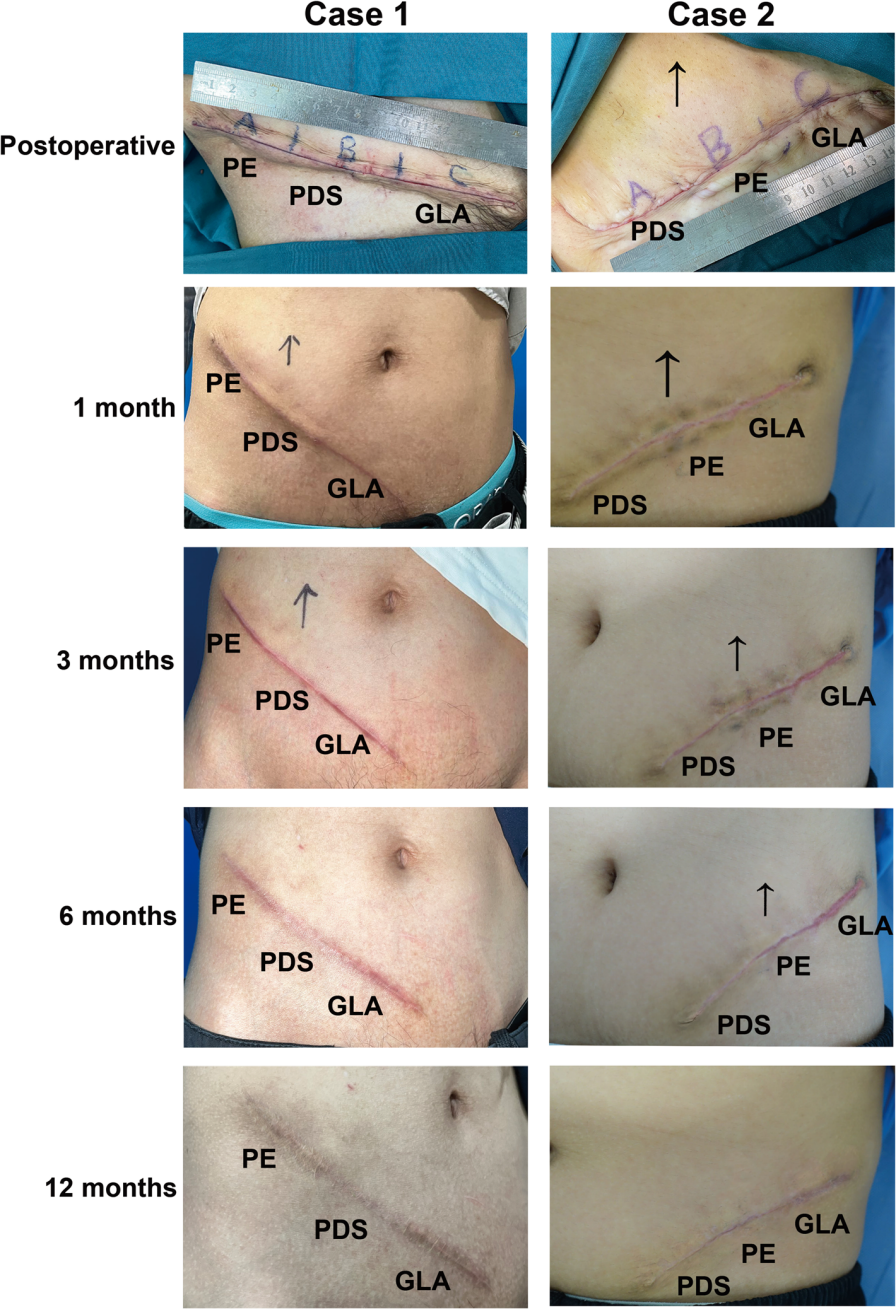
Representative wound/scar photographs at different time points: postoperatively, as well as 1-, 3-, 6- and 12-month follow-up visit. In Case 1, the parts A, B and C were sutured by PE, PDS and GLA, respectively. And in Case 2, the parts A, B and C were sutured by PDS, PE and GLA, respectively. *PE* polyester; *GLA* polyglactin; *PDS* polydioxanone

The incision was evenly divided into three segments, named A, B and C. The three different sutures were numbered 1, 2 and 3 for GLA (Polyglactin 910; Johnson & Johnson International, USA), PDS (polydioxanone; Johnson & Johnson International, USA) and PE (polyester; Johnson & Johnson International, USA), respectively. The selection of sutures used in each segment was decided by the random number (1–3 without repetition) generated by SAS (V.9.4) statistical software. The allocation assignment for participant randomization was made in a list paired with the recruitment order (Fig. 3).

### Interventions

**Fig. 4:**
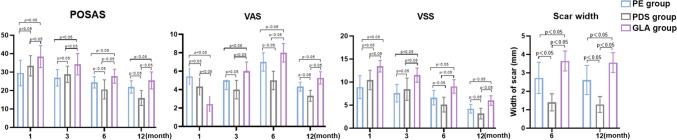
PDS brought better cosmetic scar compared to GLA or PE. Scores of VSS, VAS and POSAS in three groups at different time points, respectively. Comparison graphs of three scores *PE* polyester; *GLA* polyglactin; *PDS* polydioxanone

Patients were placed under general anesthesia, and experienced surgeons harvested free skin grafts from the lower abdomen using a standard technique. The skin around the incision was dissociated from the bottom, followed by measuring the length of the wound and dividing it into three segments evenly (Fig. 4).

Finally, each segment randomly received 2–0 GLA sutures, 2–0 PDS sutures or 2–0 PE sutures to close the incision with a WE-MBVMS. The epidermis was sutured with 6–0 monofilament threads (nylon; Covidien; USA) for the whole incision.

### Data Collection

At each follow-up point as well as at baseline, both researchers and participants were required to complete the questionnaires. In a feasible trial, the appropriate scale for measurement of the primary outcome will be selected for future full-scale RCTs. The scales used in the feasibility trial are the Vancouver Scar Scale (VSS), Visual Analog Scale (VAS) and Patient and Observer Scar Assessment Scale (POSAS).

The VSS is composed of vascularity, pigmentation, pliability and height, each of which has its own ranked subscales. By summarizing all the subscales, we can obtain a total score ranging from 0 to 13, with a higher number indicating a worse scar [9]. A previous study demonstrated that VSS is feasible for assessing cosmetic outcomes and overall satisfaction with scars [10]. As a 10-cm line with 0 representing the worst and 10 representing the best overall satisfaction, the VAS score is collected by patients placing a vertical mark on the VAS [10]. The POSAS is divided into two scales, the Patient Scar Assessment Scale (PSAS) and the Observer Scar Assessment Scale (OSAS), which are scored by patients and observers, respectively. Six domains, pain, itching, color, pliability, thickness and relief, are included in the PSAS. Six different domains are set in the OSAS, including vascularity, pigmentation, thickness, relief, pliability and surface area [11]. All items in the PSAS and OSAS are graded from 1 to 10, with higher scores suggesting a worse scar. The POSAS score is identified as the summary of scores from all items.

### Qualitative Outcomes

To ensure the acceptability of the intervention as well as the sequential follow-up, we conducted qualitative interviews with the participants. Using topics guiding the responses to scars as well as acceptance of the randomization procedure, data collection and follow-up, we concentrated on a series of information to help the smooth conduction of this research.

### Data Management

We strictly followed the standard data collection methods and employed case report forms (CRFs) as well as electronic data capture (ResMan online, http://www.medresman.org/uc/index.aspx) to record all data, including patients’ baseline information, intervention procedures and results. In the CRF, the patient’s name is identified by the initials and sex to ensure anonymity. All records were recognized via the patient’s initials, sex and ID number. Two researchers logged the data independently, and the data manager checked the records to reduce the error rate and ensure the integrity of the data.

### Statistical Analyses

With respect to the recommendations of the GCP for analyzing feasibility studies 26, we calculated descriptive statistics for feasibility outcomes, including follow-up rates, recruitment rates, interval times and baseline characteristics. Similarly, descriptive statistics were employed for the complication rate during the follow-up period. The Student’s *t* test was used to compare 2 groups, and one-way ANOVA and Fisher’s least significant difference *t* test (LSD t test) were used to compare ≥ 3 groups. GraphPad Prism (version 5.0) software was used for the above statistical analyses. *P* < 0.05 was considered to indicate statistical significance.

### Follow-up

Over time, the recruitment rate decreased. Previous research revealed that the scores of scars at 3 months were strongly positively correlated with the scores at 12 months [12]. Similarly, previous studies revealed that the cosmetic outcome of scars was already detected 3 months after suturing [7, 12–15]. In addition, the time required for complete absorption of GLA , one of the absorbable sutures we used, was 3 months, and the time required for complete absorption of PDS, the other absorbable suture we used, was 6 months [5, 16]; this means that the differences caused by sutures with different degradation times gradually becomes evident, and the effect of tension reduction tends to disappear. Therefore, we chose 3 and 6 months as the follow-up points. To better observe the change in scars over time, we set the first follow-up at 1 month and continued the follow-up to 12 months.

During the entire follow-up period, comprehensive care was provided, including but not limited to the treatment of complications. The wound data were obtained, and the appearance of the incision was recorded for the first time immediately after surgery. At each follow-up point, one professional questionnaire administrator guided the patient and two blinded observers to complete the scales measuring the scar, and digital photographs were used to record the appearance of the scar.

### Sample Size

As this is a feasibility study, formal power calculations were not carried out. Thus, we referred to similar clinical trials to determine the sample size. To detect a difference of 5 (*β* = 20%) on the 60-point POSAS with a significance level (*α*) of 0.05 and an SD of 7, we calculated that 32 patients per group were needed using GPower software V.3.1. Assuming a predicted dropout rate of 20%, 39 patients per group were required Therefore, we recruited 24 subjects.

### Patient and Public Involvement

Neither the patients nor the public were involved in the design, conduct, reporting or dissemination of this study.

### Monitoring

Adverse events were monitored and recorded using the routine reporting system by the data management committee. The Research Ethics Committee of The First Affiliated Hospital of Xi’an Jiaotong University monitored written informed consent documents, recruitment status and overall trial progress.

### Wound Suturing Using Three Different Sutures in Mice

A total of 8 8-week-old mice, 4 males and 4 females, were given free access to water and food at room temperature (22 ± 1 °C) with a humidity of 40 ± 10% and a light cycle of 12/12 h. Under aseptic conditions, the mice were treated with 10% chloral hydrate (3.0 ml/kg), followed by the creation of a full-thickness wound. The wound was evenly divided into three segments, which were randomly sutured using the three different sutures. The three sutures used were GLA (polyglactin 910; Johnson & Johnson International, USA), PDS (polydioxanone; Johnson & Johnson International, USA) and PE (polyester; Johnson & Johnson International, USA). On the 7th day, under anesthesia, we harvested the whole skin from the wound on the back followed by suturing. The whole wound tissues were fixed with 4% paraformaldehyde, embedded in paraffin, and subjected to IHC staining for IL6 and TNF-α expression. The animal experiments were approved by the institutional review board of the First Affiliated Hospital of Xi’an Jiaotong University.

## Results

Twenty-four patients were screened to participate in this study, and 18 were ultimately enrolled. Overall, 12 patients (66.7%) were male, and 6 (33.3%) were female, with ages ranging from 18 to −68 years and a median age of 34.5 years.

At 3 months after the operation, all the evaluation indices of the PE and PDS groups were better than those of the GLA group (PE, PDS and GLA groups: VSS scores: 7.62 ± 1.914, 8.44 ± 2.406 and 11.5 ± 1.543, respectively; VAS scores: 4.7 ± 1.10, 4.05 ± 0.824 and 6.2 ± 0.786, respectively; POSAS scores: 26.9 ± 4.465, 28.8 ± 4.422 and 34.2 ± 5.798, respectively, *p* < 0.05). However, at 6 months after the operation, the evaluation indices in the PDS group were better than those in the other two groups (PE, PDS and GLA groups: VSS scores: 6.6 ± 1.572, 5.1 ± 1.560 and 9.1 ± 1.474, respectively; VAS scores: 7.0 ± 0.539, 5.2 ± 0.574 and 8.0 ± 0.686, respectively; POSAS scores: 24.4 ± 4.119, 20.6 ± 5.203 and 27.7 ± 4.563, respectively; *p* < 0.05). Consistent with the results at 6 months, the PDS group still exhibited the strongest anti-scar effect at 12 months (Table 1). Moreover, the width of the scar was significantly lower in the PDS group than in the other groups at both 6 months and 12 months (PE, PDS and GLA groups: 6 months: 2.7 ± 0.822, 1.4 ± 0.450 and 3.6 ± 0.526, respectively; 12 months: 2.6 ± 0.768, 1.3 ± 0.418 and 3.6 ± 0.522, respectively) (Table 2). No significant difference was observed in the incidence of redness, swelling, or infection among the three groups at 1, 3, 6 and 12 months after the operation (*p* > 0.05). No complications occurred during the follow-up period.

**Table 1:** Postoperative scores in three groups

Time	Suture	Score		
		PE	PDS	GLA
1 month	VAS	5.4 ± 0.855	4.3 ± 0.907	2.4 ± 0.783
	VSS	8.89 ± 2.494	10.4 ± 2.121	13.4 ± 1.247
	POSAS	29.56 ± 6.94	33.39 ± 5.50	38.39 ± 6.01
	*N*	18	18	18
3 months	VAS	4.7 ± 1.100	4.06 ± 0.848	6.2 ± 0.786
	VSS	7.62 ± 1.914	8.44 ± 2.406	11.5 ± 1.543
	POSAS	26.9 ± 4.465	28.8 ± 4.422	34.2 ± 5.798
	*N*	18	18	18
6 months	VAS	7.0 ± 0.539	5.2 ± 0.574	8.0 ± 0.686
	VSS	6.3 ± 1.572	5.7 ± 1.994	9.1 ± 1.474
	POSAS	23.4 ± 4.119	20.6 ± 5.203	27.0 ± 4.563
	*N*	18	18	18
12 months	VAS	4.3 ± 0.471	3.3 ± 0.577	5.3 ± 0.650
	VSS	4.0 ± 0.943	3.5 ± 1.384	6.0 ± 1.00
	POSAS	21.9 ± 3.240	15.9 ± 3.392	25.4 ± 4.475
	*N*	18	18	18

**Table 2:** Postoperative scar width in three groups

Time score	Suture		
	PE	PDS	GLA
6 months	2.7 ± 0.822	1.4 ± 0.445	3.6 ± 0.526
12 months	2.61 ± 0.768	1.28 ± 0.418	3.56 ± 0.522

**Fig. 5:**
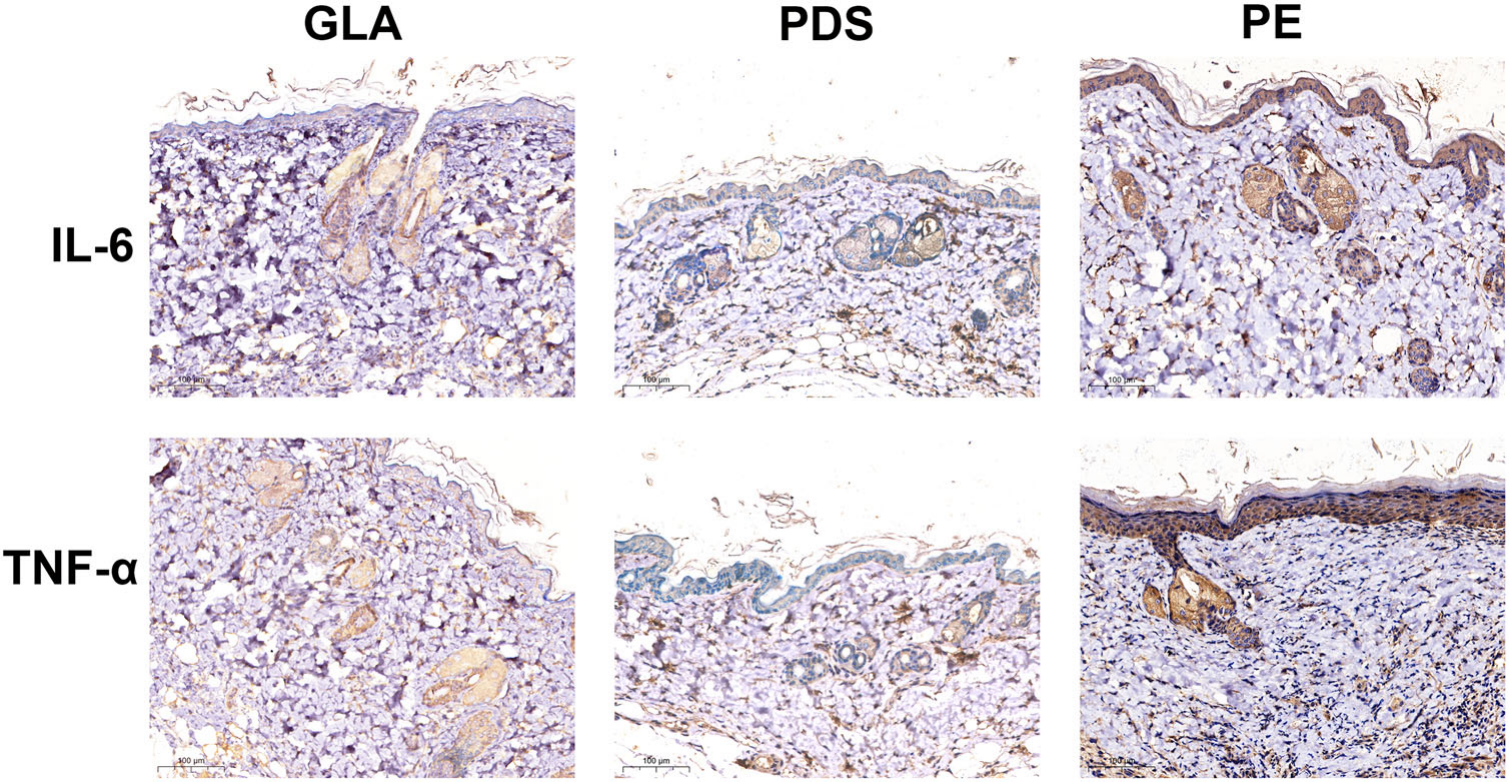
PE group showed significantly increased expression of IL-6 and TNF-α in the scar tissues. Full-thickness cutaneous wound was divided into three equilong segments previous to sutured with GLA, PE and PDS randomly. The sutures were dismantled at the seventh days and the tissues around the wound were harvested. Representative images of IHC assays in scars induced by three different sutures were performed with antibodies against IL-6 and TNF-α. *PE* polyester; *GLA* polyglactin; *PDS* polydioxanone

In our primary hypothesis, longer tension reduction leads to better cosmetic results for scars. Thus, it is not unexpected that compared with GLA, PDS results in better cosmetic scarring. The GLA is absorbed early after surgery, thus causing disappointing scar-preventing efficiency. We were always curious about the aesthetic results associated with PE and PDS. After 6 months, the PDS group had better aesthetic outcome than the PE group. We were uncertain about the underlying mechanisms; thus, we repeated the above operation on the mice. One incision was sutured using the above three sutures, and the scar tissue was harvested after 1 month. Increased expression of IL-6 and INF-α was found in tissues treated with PE (Fig. 5). These results indicated enhanced inflammation in PE-sutured tissues, which was widely demonstrated to synergically play key roles with tension to promote scar formation [17, 18].

## Discussion

In general, postoperative scarring, a sort of aesthetically undesirable complication, can hinder patients from undergoing skin-cut surgeries. Hence, any improvement in preventing scars is worthwhile. Satisfactory wound suturing is crucial for preventing scarring [19, 20]. Currently, surgeons and academicians are focused on improving suture techniques [20, 21]. Regardless of the suturing methods used, reducing the local tension is the core aim of these techniques. Additionally, it is widely accepted that reducing local tension for a sufficient amount of time is the key point in the aesthetic outcome of scars. In addition to the suture technique, the tension maintenance time and, subsequently, the aesthetic outcome of the scar are largely affected by the number of sutures used during wound closure. However, surgeons currently choose the appropriate suture material based on their experience. Studies on the aesthetic outcomes of scars caused by suture materials with different degradation durations are limited. Thus, we compared the cosmetic results of sutures with different absorbing times using a split-scar model, which showed that PDS could achieve better scar-suppressing efficiency than GLA or PE.

Using the WE-MBVMS technique, we reduced the tension and reserved tension for a certain time to confront the consistently existing tension. The suture material used in WE-MBVMS largely determines the tension reservation time, which is crucial in suppressing scarring. Studies have suggested that consistent tension reduction for 6 months is needed for an ideal cosmetic result of a scar [21, 22]. The above information led us to explore the ideal suture material for satisfactory scar prevention. In our clinical observations, PDS resulted in more slight scars than GLA. The reason for better scar suppression might be the slower degradation rate and longer tension holding time of PDS, which led us to wonder whether PE could improve the aesthetic outcome of scars. After 3 months, when PDS was partly absorbed by the human body and lost its ability to reduce tension, PE was still present and might still have the ability to resist local tension. Surprisingly, both PDS and PE had similar effects on scar suppression at 3 months, whereas PDS had a much better aesthetic effect at 6 months. To further investigate the underlying mechanism, we sutured wounds with GLA, PDS and PE using a split-scar model in mice, which revealed increased inflammation with PE treatment compared with that of PDS or GLA. Although PE is nonabsorbable and might provide a longer tension holding time, the accompanying persistent inflammation destroyed the scar suppression efficiency contributed by tension reduction.

As one of the determining factors in wound healing and scar formation, inflammation has dramatic impacts on the pathogenesis of scars [23]. Inflammation is triggered immediately after injury with cascades of reactions of immune cells, including macrophages and neutrophils, and subsides to a certain level approximately 72 h later [24]. The process of wound healing and scar formation requires the regulation of numerous cellular populations and intricate synchronization, during which inflammation occurs [25]. Previous studies have demonstrated a positive correlation between inflammation and the final scar size [18, 26, 27]. Mechanically, inflammation contributes to fibroblast survival and stimulates the transformation of fibroblasts into myofibroblasts, which is attributed to the abundance of inflammatory cytokines that are consequently associated with positive modulation of collagen synthesis [28, 29]. In our study, upregulated IL-6 and TNF-α were found with PE treatment, which illustrated the reason for unsatisfactory scar formation.

Compared with PDS and PE, GLA causes the worst scar-preventing outcome, possibly because of its short tension holding time, which is only 30 days. A sufficient duration of tension reduction, which is suggested to be longer than 6 months, is important for scar prevention [30]. Thus, in cases where subcutaneous GLA sutures are used for tension-reducing closure, prolonged postoperative use of Steri-Strips (for more than 3 months) may contribute to decreased wound tension, which in turn may help to inhibit hypertrophic scarring. Our results suggested that although PE-induced inflammation attenuated scar prevention, its ability to reduce tension adequately offset adverse effects and ultimately resulted in better aesthetic results than GLA. This result demonstrated the important role of tension reduction in scar suppression.

In this study, we used a trisection-split model to compare the cosmetic results of the different suture materials, which may help minimize confounders. Moreover, we estimated the results of the surgery in the same field of view, which is simpler and more intuitive than having to compare interventions between different patients for the observer. The trisection-split model has been used in numerous surgical studies to compare different techniques and has been proved to be reliable and feasible [31, 32].

After 1, 3, 6 and 12 months, validated scar assessment scales were used to rate the results. To increase the value of the scoring assessments of the results, the patient and the observer were blinded to the treatments. In this trial, we found that PDS ultimately provided the best postoperative cosmetic results. However, no statistically significant difference was found in the degree of wound eversion postoperatively provided by the three sutures, which means that the benefit of wound eversion is controversial. Hence, we assume that the different results of the three sutures might be mainly related to the wound tension provided by the sutures, which has also been mentioned by other researchers^32-34^.

During the follow-up period, no wound complications occurred, which might be related to the small sample size. Despite the 12-month follow-up in this study, it is unclear whether follow-up at only 3 or 6 months postoperatively would be adequate to capture the full extent of the anti-scar efficacy. Some similar comparative studies have shown that differences in scars can be observed at 3 months after intervention. Moreover, researchers also found no differences in the evaluation of scars between assessments performed at 6 months and 1 year [7, 14, 15]. In addition, an excessive follow-up period could increase the patient dropout rate and decrease the quality of the study. Certainly, the shortest follow-up duration sufficient for assessing scar inhibition remains unclear and deserves further study.

## Conclusion

Sufficient tension holding time is a very important factor for inhibiting scar formation. Meanwhile, we should focus attention on inflammation in the local microenvironment.

## Data Availability

Jing Jia and Maoguo Shu had full access to all data in the study and accepted responsibility for the integrity of the data and the accuracy of the data analysis.

## Declarations

### Trial Registration Number

The study is registered with ClinicalTrials.gov (NCT04941911).
